# Disruption of *tp53* leads to cutaneous nevus and melanoma formation in *Xenopus tropicalis*

**DOI:** 10.1002/1878-0261.13301

**Published:** 2022-08-18

**Authors:** Rensen Ran, Lanxin Li, Zhaoying Shi, Guanghui Liu, Hao Jiang, Liangchen Fang, Tingting Xu, Jixuan Huang, Weiqi Chen, Yonglong Chen

**Affiliations:** ^1^ School of Life Science and Technology Harbin Institute of Technology China; ^2^ Department of Biology, Guangdong Provincial Key Laboratory of Cell Microenvironment and Disease Research, Shenzhen Key Laboratory of Cell Microenvironment, School of Life Sciences Southern University of Science and Technology Shenzhen China; ^3^ School of Medical Technology and Engineering Fujian Medical University Fuzhou China

**Keywords:** Li–Fraumeni syndrome, melanoma, nevi, *tp53*, *Xenopus tropicalis*

## Abstract

In humans, germline *TP53* mutations predispose carriers to a wide spectrum of cancers, which is known as Li–Fraumeni syndrome (LFS). To date, the association of melanomas with LFS remains unestablished. No melanomas have been reported in any P53‐modified mouse models either. In this study, we show that targeted disruption of P53 at the DNA‐binding domain in *Xenopus tropicalis* recapitulates LFS, with the formation of soft‐tissue sarcomas and pancreatic ductal adenocarcinoma. Interestingly, 19% of the 14‐month‐old *tp53* ^*Δ7/Δ7*^ homozygotes and 18% of *tp53* ^*+/Δ7*^ heterozygotes spontaneously developed small nevi and non‐invasive melanomas. Large invasive melanomas were also observed in other older homozygous mutants, with about 7.9% penetrance. Our data suggest that more dermatologic investigation of LFS patients should be able to settle the association of melanoma with LFS in epidemiology. Our model is also valuable for further investigation of the molecular mechanism underlying melanoma progression upon germline alteration of the *tp53* locus.

## Graphical abstract

In humans, the association of melanomas and germline *TP53* mutations remains unsettled. Melanomas have not been observed in any *Trp53*‐modified mouse models either. Here, we show that targeted disruption of P53 at the DNA binding domain in *Xenopus tropicalis* led to nevus and melanoma formation, which recapitulates the process of human cutaneous melanoma development.

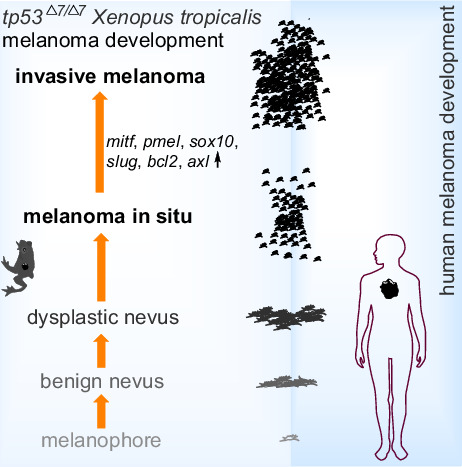

**Abbreviations**
H&E: Hematoxylin–Eosin
HCG: human chorionic gonadotropin
LFS: Li–Fraumeni syndrome
LOH: loss of heterozygosity
SD: standard deviation
WT: wild‐type

## 1 Introduction

The full‐length canonical P53 (also named P53α, FLP53, TAP53α), encoded by *TP53* gene in human, contains two transactivation domains, a conserved proline‐rich domain, a central DNA‐binding domain, a nuclear localization signal, and an oligomerization domain in succession from N terminus to C terminus. To date, nine *TP53* mRNAs are found in humans resulted from alternative promoter usage (promoter 1 and promoter 2) at the *TP53* locus and alternative splicing of intron 2 and intron 9, which further encode 12 different P53 protein isoforms via translation initiation at different start codons [1]. Various functional P53 isoforms are also described in *Drosophila*, zebrafish, and mice [1, 2, 3]. Although the whole landscape of P53 isoform network in human physiology, cancer, and degenerative diseases remains largely unknown, accumulated evidence based on clinical studies as well as studies with cell lines and animal models supports the notion that depending on the cellular or tissue context, P53 isoforms are differentially co‐expressed and work in concert to define cellular responses, suggesting the complication in deciphering the consequence of any alterations at the *TP53* locus [3]. Indeed, it is difficult in the clinic to link *TP53* mutation status to cancer treatment and prognosis and *TP53* is unfortunately the most frequently mutated gene in human cancers [2, 3, 4].

Germline *TP53* alterations predispose carriers to a wide spectrum of cancers known as the Li–Fraumeni syndrome (LFS) [5]. Given the intricate P53 isoform network, interpretation of germline *TP53* variants is particularly challenging, which needs the integration of epidemiological, phenotypical, bioinformatics prediction, and functional data [6]. The majority of the germline *TP53* mutations validated so far are missense mutations and occur in the highly conserved DNA‐binding domain (https://tp53.isb‐cgc.org/). The penetrance of germline disease‐causing *TP53* variants is variable depending on the type of variants, tissue‐specific expression of P53 isoforms, context‐dependent genomic and epigenetic comodifiers, and context‐dependent stress stimuli, thus showing apparent phenotypic heterogeneity. The five core cancers most commonly observed in LFS are breast cancer, soft tissue sarcomas, brain tumors, adrenocortical carcinomas, and bone sarcomas [5, 6]. In terms of epidemiological analysis, rare cases of melanoma reported in LFS patients are very inconclusive and additional data are crucially needed [7].

In mice, the P53 encoding gene is named as *Trp53*. Mice with a germline *Trp53* partial deletion are developmentally normal and fertile, yet highly susceptible to T‐cell lymphomas and sarcomas [8]. A mechanistic study demonstrates that the initial inherited loss of P53 functions in mice delineates an order of genetic alterations, including *Pten* deletion, Cyclin D–Cdk6 overexpression, and Ikaros splicing changes, which are selected for in an oligoclonal selection fashion during the evolution of these thymic lymphomas [9]. Genetic background also plays an important role in the predisposition of tumor types [10]. C57BL6 mice have intrinsic resistance to mammary carcinomas. Backcross of the C57BL6 *Trp53*‐null allele onto the BALB/c background resulted in 55% of the female BALB/c *Trp53* ^*+/−*^ mice to present with mammary carcinomas [11]. Elegant genotype–phenotype (knock‐in) studies in mice recapitulate several human *TP53* missense mutation–associated LFSs. Together, mouse models help elucidate molecular and pathogenic mechanisms of tumorigenicity in LFS and further validate the complexity of P53‐mediated cancer predisposition. Of note, melanoma has not been reported in any of the established mouse *Trp53*‐modified models [12, 13, 14, 15].

Several types of *tp53* mutants generated in zebrafish developed a tumor spectrum characteristic of malignant peripheral nerve sheath tumors, sarcomas, angiosarcoma, germ cell tumors, and leukemia [16, 17, 18]. Preciously, a single melanoma was observed in one out of over 500 *tp53* ^−/−^ fish aged 11 months, indicating an extremely low penetrance [16]. Targeted disruption of *Xenopus tropicalis* P53 at the proline‐rich domain, which is in front of the DNA‐binding domain, led to the spontaneous formation of hematological malignancy and sarcomas. No melanoma was described in these frogs [19].

In this study, we find that on targeting the DNA‐binding domain of *X. tropicalis* P53, about 19% of homozygous mutants developed nevi and melanomas, which provides a valuable model for both the investigation of phenotypic heterogeneity predisposed by germline *TP53* alterations and the mechanistic study of P53 network‐mediated melanoma development.

## 2 Materials and methods

### 2.1 *Xenopus tropicalis* maintenance

Adult *X. tropicalis* frogs were purchased from Nasco (Fort Atkinson, WI, USA; http://www.enasco.com). Frog maintenance and husbandry was carried out following the approved laboratory practices. All *X. tropicalis* experiments were approved by the Institutional Animal Care and Use Committee at the Southern University of Science and Technology (SUSTC‐JY2018059).

### 2.2 Preparation of Cas9 mRNA and guide RNA, embryo microinjection, genotyping, and off‐target analysis

pCS2‐SpCas9 plasmid was linearized with *Not* I [20], and transcribed with the mMessage mMachine SP6 Kit (Ambion, Austin, TX, USA). *tp53* gRNA target sequence is 5′‐GGCCAAGACCTGCCCTTTGCTGG‐3′. gRNA template was generated with the pUC57‐T7‐gRNA scaffold vector by PCR amplification with the Trac‐reverse primer and a gRNA forward primer containing a T7 promoter and the gRNA target sequence [21]. Primer sequences were listed in Table S1. The *in vitro* transcription of gRNA was implemented with the Transcript Aid T7 High Yield Transcription Kit (Thermo Fisher Scientific, Rockford, IL, USA). The Cas9 mRNA and gRNA were purified with the RNeasy Mini Kit and miRNeasy Mini Kit (Qiagen, Hilden, Germany), respectively.

*Xenopus tropicalis* fertilized eggs were obtained by hormone‐induced mating. Adult male and female frogs were pre‐primed with 20 units of human chorionic gonadotropin (HCG) 1 day in advance and followed by a full dose (145 units of HCG) priming about 2–3 h before embryo collection. Cas9 mRNA (300 pg per egg) and *tp53* gRNA (200 pg per egg) were mixed and injected into fertilized eggs (2 nL per egg) from the animal pole.

To check the targeted *tp53* disruption efficiency, 24 h after microinjection, we randomly pooled five healthy embryos, extracted genomic DNA, amplified the targeted region by PCR, and then cloned the purified PCR products into the pMD18‐T vector (Takara, Katsu, Japan) by TA cloning. Single colonies were randomly picked for Sanger DNA sequencing analyses to detect any indel mutations. Mating of G0 frogs resulted in the generation of *tp53* disrupted homozygous frogs. Small pieces of nails were taken for genotyping of adult frogs. The sequences of genotyping PCR primers were listed in Table S1.

To identify potential off‐target sites, all genomic loci containing up to five mismatches compared with the coding sequence for the tp53 gRNA followed the NGG PAM sequence were identified by mapping the targeted site to *X. tropicalis* genome (JGI 9.0) using CRISPOR [22]. Based on the NCBI database, four pairs of primers (Table S1) were designed for RT‐PCR amplification of four different *X. tropicalis tp63* mRNAs for Sanger DNA sequencing analysis.

### 2.3 Loss of heterozygosity analysis

To check the genotypes of the dysplastic nevi developed in the *tp53* ^*+/Δ7*^ heterozygotes, pigmented nevus cells and neighboring stratum compactum cells were isolated by laser capture microdissection (Leica LMD7000, Wetzlar, Germany) of 20 μm thick cryosection slices of the lesion. After genomic DNA extraction, PCR products containing the Δ7 region were obtained by three independent PCR amplifications with the *tp53* genotyping primers (Table S1) and cloned into the pCE2 TA/Blunt‐Zero vector. Twenty‐five colonies from each amplification were sequenced by Sanger DNA sequencing.

### 2.4 Hematoxylin–Eosin (H&E) staining

For histological analyses, normal skin and nevus/melanoma lesions were excised and fixed in FAS eyeball fixative (Servicebio, Wuhan, China) at room temperature for 24 h. Then the fixed samples were dehydrated through 75%, 85%, 95%, and 100% ethanol, subsequently replaced with xylene, and embedded in paraffin wax. Sections of 6 μm thickness were dewaxed in xylene and rehydrated through 100%, 95% and 70% ethanol before being stained with the H&E staining Kit (Baso, Zhuhai, China). The stained sections were imaged with an Olympus bx53 upright microscope (Olympus, Tokyo, Japan).

### 2.5 X‐ray irradiation, western blot analysis, and RT‐PCR analysis

*Xenopus tropicalis* stage 10 embryos with different genotypes were irradiated with serial doses of X‐ray using an RS2000 X‐ray irradiator (Rad Source, Buford, GA, USA) and their survival rates were evaluated during subsequent development. For western blot analyses, Anti‐P53[X77] (ab16465; Abcam, Cambridge, UK) antibody was used to detect wild‐type P53 in *X. tropicalis*, and anti‐β‐tubulin (ab6046; Abcam) antibody was used as a loading control. Goat anti‐rabbit or goat anti‐mouse HRP‐conjugated (HS101‐01 and HS201‐01; Transgen Biotech, Beijing, China) antibodies were used as secondary antibodies. For RT‐PCR analyses, total RNA was extracted with the TransZol Up Plus RNA Kit (ER501‐01; Transgen Biotech). cDNA was prepared with the *TransScript*® Uni One‐Step gDNA Removal and cDNA Synthesis SuperMix (AU311‐03; Transgen Biotech). The sequences of RT‐PCR primers are listed in Table S1.

## 3 Results

### 3.1 Establishment of a *tp53* disrupted *Xenopus tropicalis* line

**Fig. 1:**
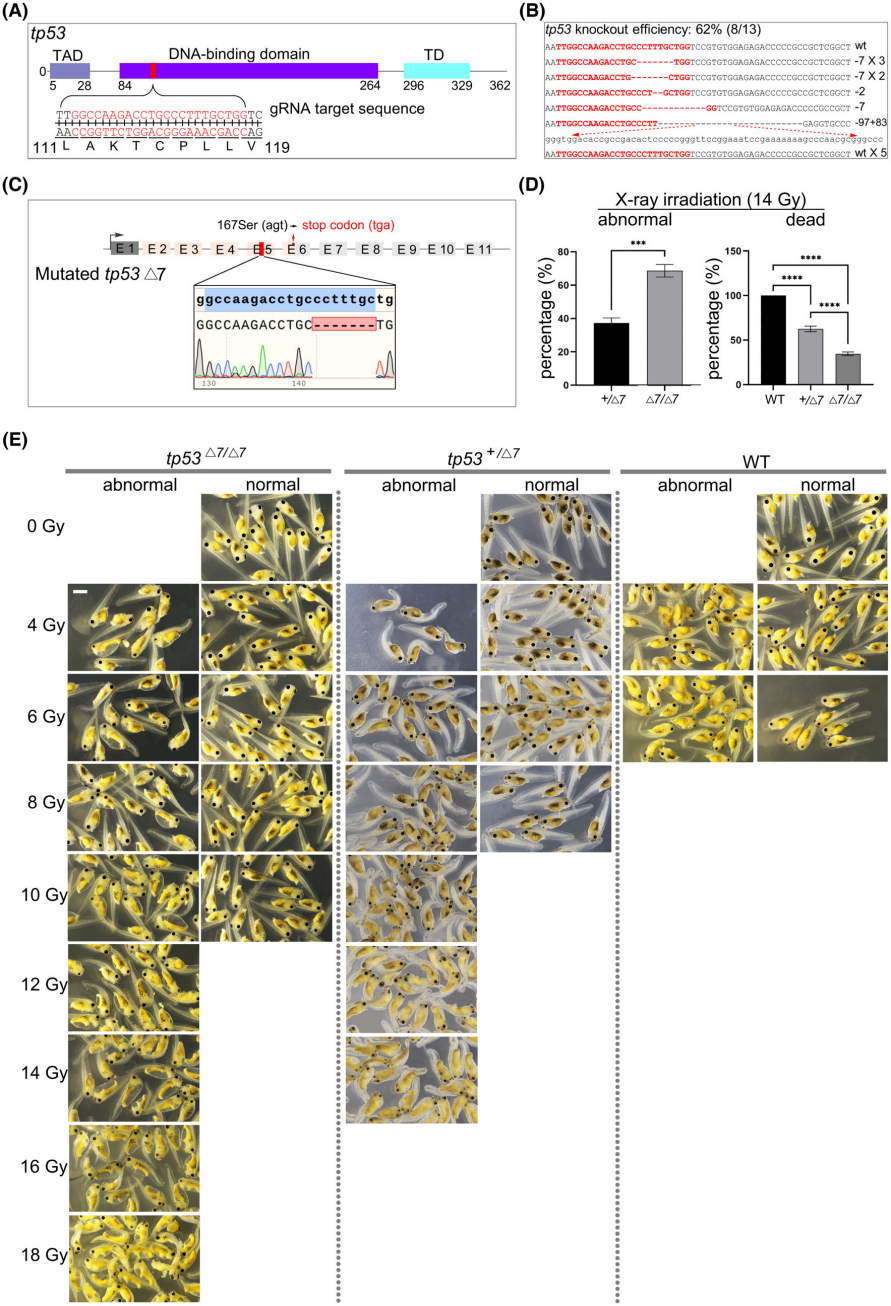
Establishment of the *Xenopus tropicalis tp53* Δ7 mutant line. (A) gRNA design. TAD, transactivation domain; TD, tetramerization domain. (B) Sanger DNA sequencing data show mutations induced by targeting *X. tropicalis tp53*. The wild‐type (wt) sequence is shown at the top with the target site in red. Red dashes indicate deletions and lowercase letters indicate insertions. (C) The *tp53* Δ7 genotype in the F1 offspring. E, exon. Seven out of 29 F1 adults showed this Δ7‐heterozygous genotype. (D) Histograms of statistical data (mean values ± SD) from three independent X‐ray irradiation (14 Gy) experiments show distribution of abnormal and dead embryos among three genotypes. WT, wild‐type. graphpad prism 9 (GraphPad Software, San Diego, CA, USA) was used for the *t*‐test (***P < 0.001, ****P < 0.0001). (E) Representative images from one experiment show that P53‐dependent radiosensitivity in WT *X. tropicalis* embryos was proportionally decreased in *tp53* ^*+/Δ7*^ and *tp53* ^*Δ7/Δ7*^ mutants. Identical results were obtained in three independent experiments, with 9216 embryos used in total. All the embryos were irradiated at stage 10 and evaluated at stage 41. In a dose‐dependent manner, no wild‐type embryos can survive to stage 41 at 8 Gy irradiation. In contrast, *tp53* ^*+/Δ7*^ heterozygotes and *tp53* ^*Δ7/Δ7*^ homozygotes can survive to stage 41 even at 14 and 18 Gy irradiation, respectively (for statistics, see Table S2). Scale bar, 0.5 mm.

Given that the majority of the germline *TP53* mutations occur in the highly conserved DNA‐binding domain in humans, we chose to design a gRNA targeting *X. tropicalis tp53* DNA‐binding domain coding region for Leu111 to Val119 (Fig. 1A). The targeting efficiency is about 62% (Fig. 1B). By mating of the sexually mature G0 mosaic founders, we were able to establish a *X. tropicalis tp53* Δ7 mutant line, of which both the *tp53* ^*+/Δ7*^ heterozygotes and the *tp53* ^*Δ7/Δ7*^ homozygotes developed normally and were fertile. The frame‐shifting Δ7 deletion causes a premature stop codon *tp53* ^*167STOP*^ to the canonical full‐length *tp53* mRNA (Fig. 1C). Upon X‐ray irradiation that was used for detecting canonical P53‐dependent radiosensitivity in mouse embryos [23], we observed mild and severe reduction of radiosensitivity in *tp53* ^*+/Δ7*^ heterozygotes and the *tp53* ^*Δ7/Δ7*^ homozygotes, respectively, indicating a functional truncation of canonical full‐length P53 in these frog mutants (Fig. 1D,E and Table S2).

**Fig. 2:**
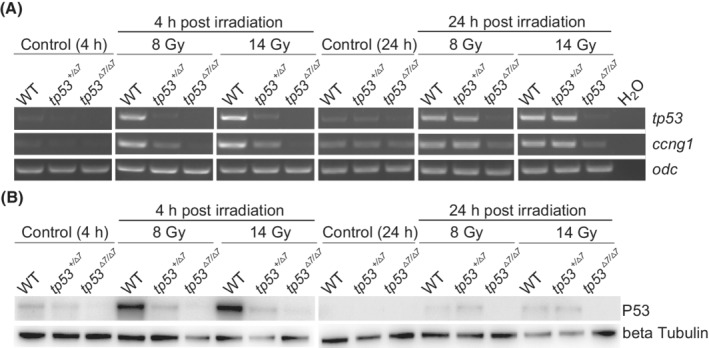
P53 haploinsufficiency in *Xenopus tropicalis tp53* ^*+/Δ7*^ mutants. (A) RT‐PCR analysis of *tp53* and *ccng1* expression in samples indicated in the image. *odc* was used as a RNA loading control. Identical results were obtained in three independent experiments. (B) Western blot analysis shows P53 levels in samples as indicated. Beta tubulin was used as a protein loading control. h, hour; WT, wild‐type. This experiment was carried out once.

Upon irradiation, an early (4 h post‐irradiation) compromised transcriptional activation of *tp53* and its target gene *ccng1* in heterozygotes was revealed by RT‐PCR analysis, which was compensated later (24 h post‐irradiation) probably due to mRNA accumulation (Fig. 2A). A low level of *ccng1* transcript detected in all tailbud stage *tp53* ^*Δ7/Δ7*^ embryos as well as in non‐irradiated wild‐type and *tp53* ^*+/Δ7*^ embryos at tailbud stage of development suggests a P53‐independent basal activation of *ccng1* transcription as development proceeded (Fig. 2A). Consistently, western blot analysis revealed an acute and drastic stabilization of canonical full‐length P53 protein in wild‐type embryos 4 h post‐irradiation, which in 24 h returned to a low level similar to that in un‐irradiated wild‐type gastrula embryos, indicating relatively rapid P53 protein homeostasis likely regulated by a negative feedback (Fig. 2B). Of note, the full‐length P53 protein in wild‐type embryos decreased to an undetectable level at tailbud stage of development, which is reminiscent of the data from a previous study [24]. In contrast, the acute stabilization of P53 protein was severely compromised in *tp53* ^*+/Δ7*^ heterozygotes 4 h post‐irradiation and became indistinguishable from that in wild‐type embryos 24 h post‐irradiation (Fig. 2B). In gastrula stage embryos without irradiation, *tp53* ^*+/Δ7*^ heterozygotes appear to have about half amount of P53 protein in wild‐type siblings. As expected, full‐length P53 was undetectable in any *tp53* ^*Δ7/Δ7*^mutants (Fig. 2B). Thus, the intermediate phenotype of *tp53* ^*+/Δ7*^ heterozygotes in response to X‐ray irradiation is likely due to canonical P53 haploinsufficiency. Collectively, these results indicate that we establish a *tp53* disrupted *X. tropicalis* line deficient of functional canonical P53.

### 3.2 Spontaneous formation of nevi and melanoma in the *tp53* ^*Δ7*^ ^*/*^ ^*Δ7*^ *Xenopus tropicalis* frogs

**Fig. 3:**
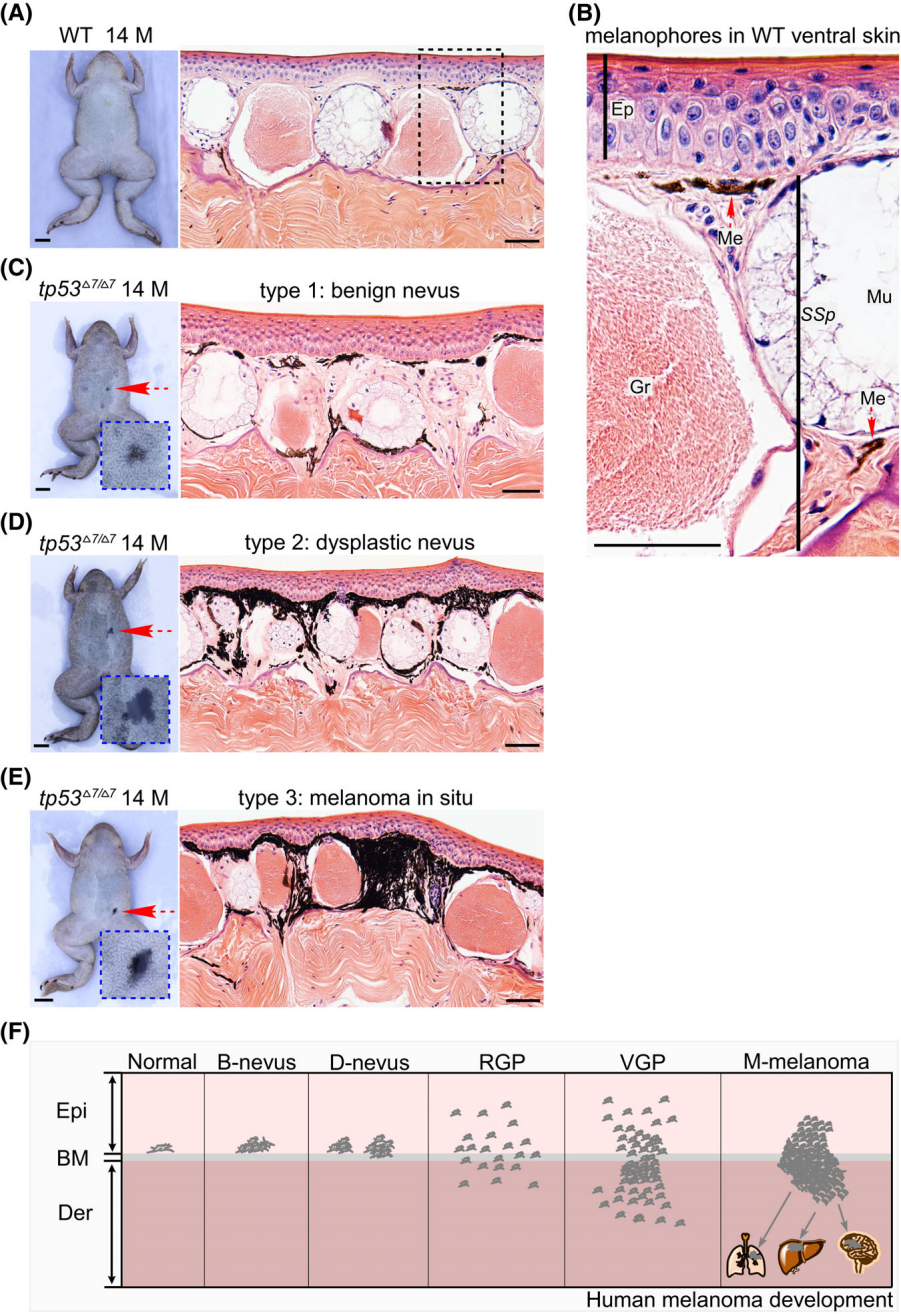
Nevus and melanoma formation in *tp53* ^*Δ7/Δ7*^ adult frogs. (A) Ventral view of a 14‐month‐old wild‐type (WT) frog shows no nevi. H&E staining indicates representative histological structure of the ventral skin. Similar structure was observed in all 50 serial sections. (B) Higher magnification view of the dashed box area in A shows that the majority of the melanophores are located just beneath the basement membrane and a small portion at the bottom of a mucous gland (red dashed arrows). Ep, epidermis; Me, melanophores; Mu, mucous glands; Gr, granular glands; *SSp*, *stratum spongiosum*. Similar structure was observed in all 50 serial sections. (C) Ventral view of a 14‐month‐old *tp53* ^*Δ7/Δ7*^ mutant shows a benign nevus (red dashed arrow and the higher magnification view in the blue dashed box). A representative H&E staining reveled local hyperproliferation of melanophores. Similar phenotype was observed in all 10 serial sections. (D) Ventral view of another 14‐month‐old *tp53* ^*Δ7/Δ7*^ mutant shows a dysplastic nevus (red dashed arrow and the higher magnification view in the blue dashed box). A representative H&E staining reveled aberrant growth of melanophores. Similar phenotype was observed in all 10 serial sections. (E) Ventral view of a 14‐month‐old *tp53* ^*Δ7/Δ7*^ mutant shows a melanoma *in situ* (red dashed arrow and the higher magnification view in the blue dashed box). A representative H&E staining revealed apparent vertical growth of melanophores across the stratum spongiosum. Similar phenotype was observed in all 10 serial sections. (F) Schematic adapted from [28] shows the histological steps in the progression of human melanoma. B, benign; BM, basement membrane; D, dysplastic; Der, dermis; Epi, epidermis; M, months; M‐melanoma, metastatic melanoma; RGP, radial growth phase; VGP, vertical growth phase; scale bars in frog images are 5 mm. Scale bars in H&E staining images are 50 μm.

During the raising of the *tp53* mutant line, we noticed that small hypermelanotic lesions started to appear in both the hetero‐ and homozygous mutant frogs by 6 months, but not in wild‐type siblings. Among 115 14‐month‐old *tp53* ^*Δ7/Δ7*^ frogs (Clutch 3), 22 (19%) displayed the nevus‐like lesions without other discernable anomalies. Four out of 22 *tp53* ^*+/Δ7*^siblings (18%) showed similar lesions. We randomly selected eight lesion‐positive homozygotes and biopsied all their lesions for histological analyses. Based on the histological data, the lesions can be categorized into three types (Fig. 3). First, it should be clarified that in wild‐type frogs, cutaneous melanophores are located mainly in the dorsal dermis, much less in the ventral dermis, and scantily in the epidermis of both the dorsal and the ventral skin [25, 26]. The dermal distribution pattern of melanophores in both the dorsal and the ventral skin is similar, with the majority just beneath the basement membrane and a small portion at the border of the stratum spongiosum and the stratum compactum (Fig. 3A,B) [25]. In sharp contrast to the wild‐type pattern, the histological features of Types 1–3 lesions clearly demonstrate patterns of melanophore hyperproliferation *in situ*, lesion radial growth mainly underneath the basement membrane, and lesion vertical growth across the mucous and serous glands within the stratum spongiosum territory, respectively (Fig. 3C–E), which are reminiscent of the histological changes accompanying human melanoma progression [27, 28]. Thus, we tentatively designate these lesions as benign nevi, dysplastic nevi, and melanoma *in situ*, respectively, which reflects a stepwise transformation of melanophores to non‐invasive melanoma. From visual inspection, these lesions became gradually darker during the transformation and occurred spontaneously without dorsoventral preference (Fig. 3 and Table 1). Of note, they were uniformly small (diameter < 1.5 mm) by 14 months (Fig. 3).

**Table 1:** The nevi and non‐invasive melanoma formation in eight *tp53* ^*Δ7/Δ7*^ *Xenopus tropicalis* aged 14 months.

*tp53* ^*Δ7/Δ7*^ frogs no.	Location	Benign nevi (16/25, 64%)	Dysplastic nevi (5/25, 20%)	Non‐invasive melanoma (4/25, 16%)
1	Dorsal	2		
	Ventral	2		
2	Dorsal			
	Ventral	2		
3	Dorsal	1	1	
	Ventral	1	1	
4	Dorsal			
	Ventral	2	1	1
5	Dorsal			
	Ventral			
6	Dorsal	1		
	Ventral		1	1
7	Dorsal	4		
	Ventral	1		
8	Dorsal		1	
	Ventral			1

**Fig. 4:**
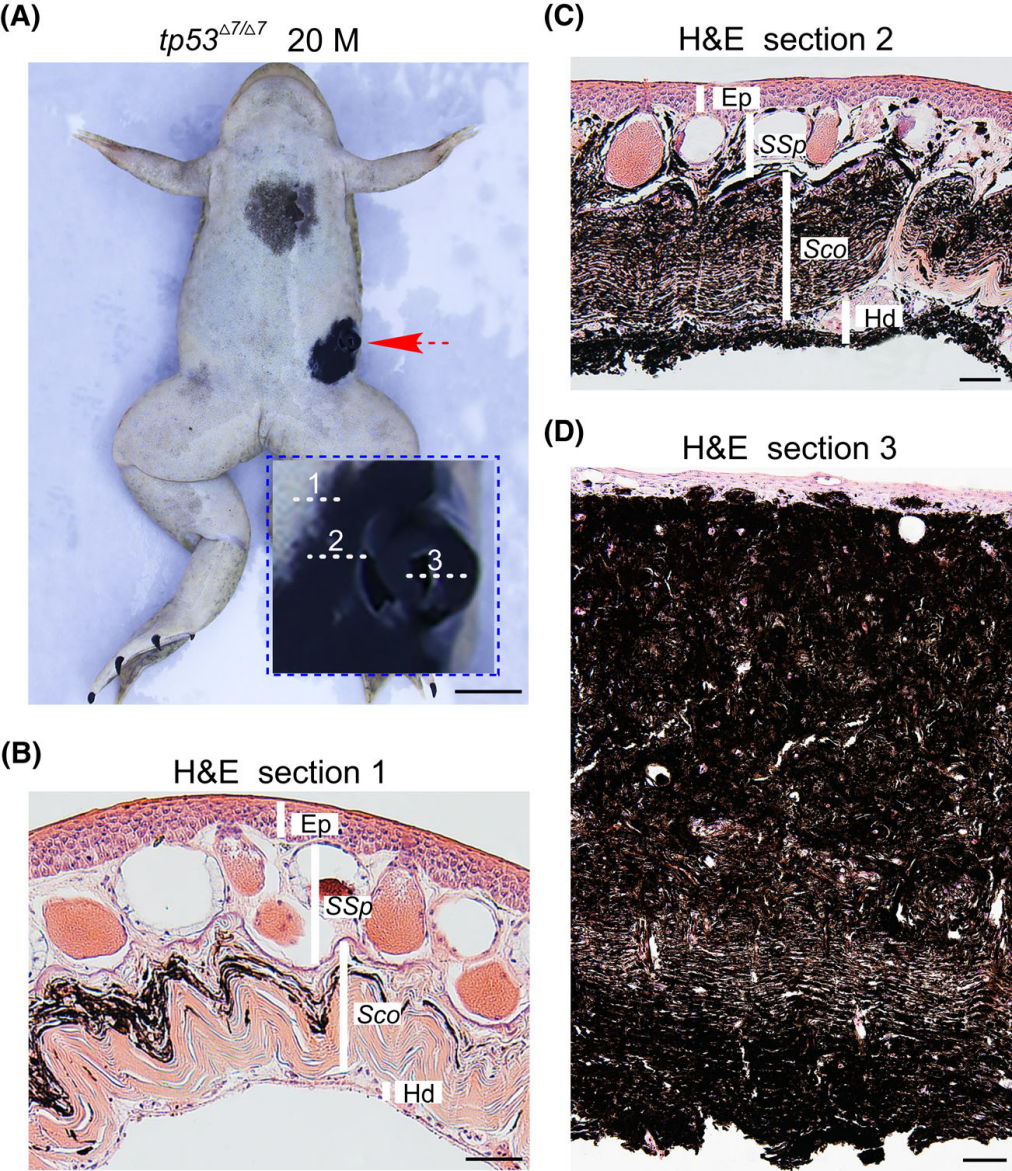
Invasive melanoma (about 5 mm) developed in a 20‐month‐old *tp53* ^*Δ7/Δ7*^ frog. (A) Ventral view of the 20‐month‐old *tp53* ^*Δ7/Δ7*^ frog shows an invasive melanoma (red dashed arrow and the higher magnification view in the blue dashed box). Scale bar, 5 mm. (B–D) H&E staining of representative sections from the area, as indicated by the dashed lines 1, 2, and 3 in A, respectively. For areas indicated by each line, similar structures were observed in at least 10 neighboring serial sections. Ep, epidermis; M, months; Hd, hypodermis; *Sco*, *stratum corneum*; *SSp*, *stratum spongiosum*. Scale bars, 50 μm.

Much larger (diameter > 5 mm) lesions appeared in two moribund frogs from two clutches of older *tp53* ^*Δ7/Δ7*^ mutants by age of 20 (Clutch 2, 21 frogs) and 34 (Clutch 1, 17 frogs) months, respectively. Histological data revealed clear cancerous melanophore invasion into the entire stratum compactum of dermis, the epidermis, and the hypodermis (Fig. 4 and Fig. S1). These lesions can be designated as an invasive melanoma, although the penetrance is low (2/38).

**Fig. 5:**
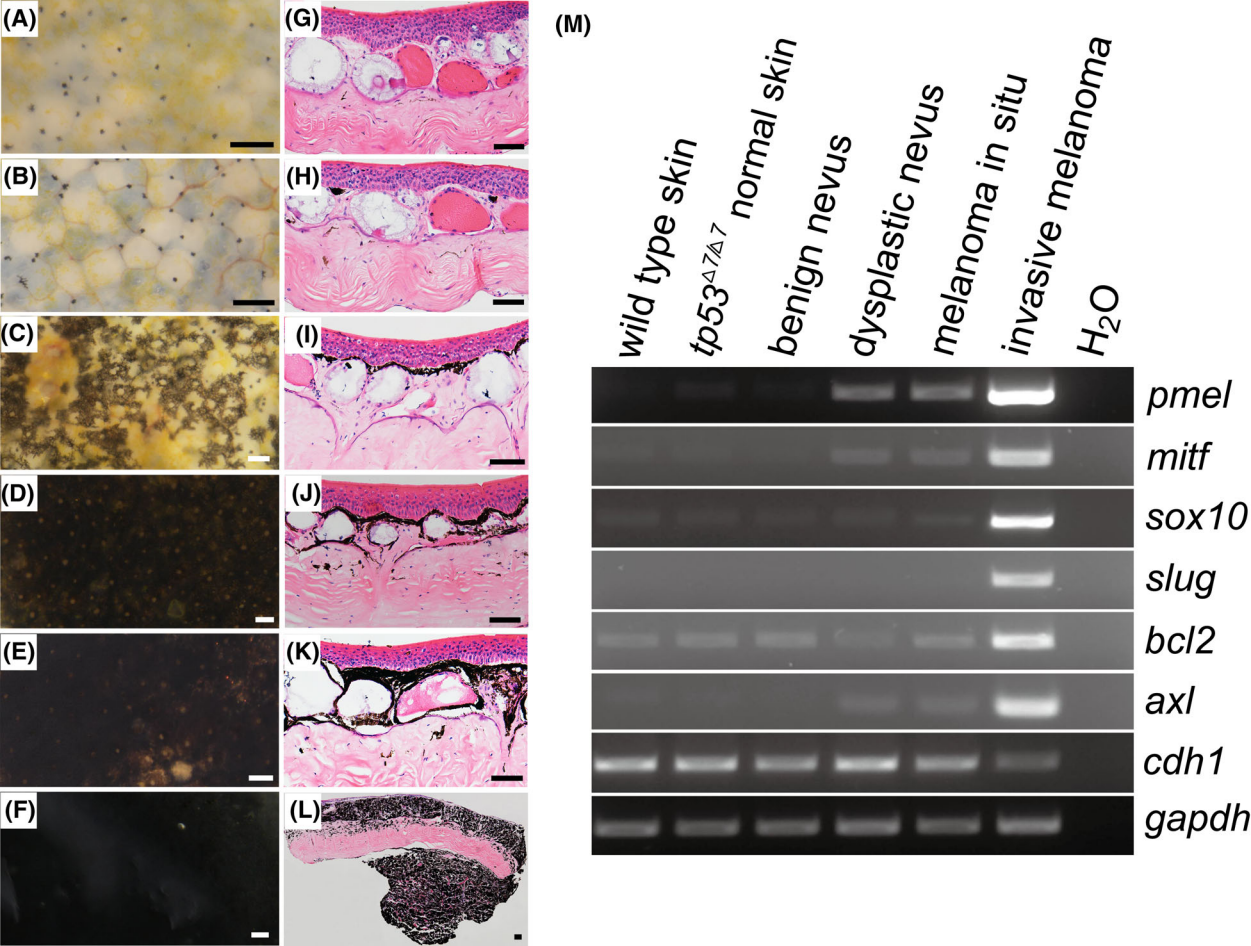
Histology‐based nevus and melanoma classification is confirmed by the expression levels of molecular marker genes. (A–F) Representative morphology of dissected skin samples under stereo microscope. For each category, two frogs were biopsied and similar morphology was observed. (A) Ventral skin sample from a 19‐month‐old wild‐type frog; (B) ventral skin sample from a 19‐month‐old *tp53* ^*Δ7/Δ7*^ frog without nevus/melanoma lesions; (C–E) ventral skin samples from 19‐month‐old *tp53* ^*Δ7/Δ7*^ frogs with benign nevus, dysplastic nevus, and melanoma *in situ*, respectively; (F) ventral skin sample from a 25‐month‐old *tp53* ^*Δ7/Δ7*^ frog with an invasive melanoma. Scale bars, 100 μm. (G–L) Representative histological structures (H&E staining) of samples shown in A–F, respectively. Similar structures were observed in 10 serial sections from each sample. Scale bars, 50 μm. (M) RT‐PCR analyses revealed the transcriptional expression levels of genes indicated on the right side in different samples listed on the top. *gapdh* was used as an RNA loading control. Identical results were obtained in two independent RT‐PCR experiments.

Given the paucity of antibody availability in frogs, to further verify our histology‐based classification of nevi and melanomas, we carried out RT‐PCR analysis instead of immunohistochemical analysis. Benign nevus, dysplastic nevus, and melanoma *in situ* samples for RNA extraction were biopsied from the Clutch 3 *tp53* ^*Δ7/Δ7*^ homozygotes, now 19‐month‐old with average diameter of about 2 mm for dysplastic nevi (Fig. S2). The invasive melanoma sample was biopsied from a relatively healthy melanoma‐harboring frog of Clutch 2 *tp53* ^*Δ7/Δ7*^ mutants, now 25‐month‐old with average diameter of about 3 mm for dysplastic nevi (Fig. S2). Each dissected lesion was first photographed using a stereo microscope (SMZ18; Nikon, Tokyo, Japan) (Fig. 5A–F) and then divided into two halves, one again for histological documentation (H&E staining) (Fig. 5G–L) and the other half for RNA extraction and RT‐PCR analysis (Fig. 5M). The graded increase of melanophore density and darkness from benign nevus to invasive melanoma was clearly visualized under the stereo microscope (Fig. 5A–F). For RT‐PCR analyses, we chose melanophore marker genes *pmel* [29] and *mitf* [30], neural crest markers *sox10* and *slug* that can be reactivated in invasive melanomas [31], melanoma‐enriched genes *bcl2* [32] and *axl* [33], and *cdh1* (E‐cadherin gene), of which expression was decreased in invasive melanomas [34]. The transcript levels of these marker genes in our samples are reminiscent of their expression levels in corresponding lesions based on previous studies [29, 30, 31, 32, 33, 34], which clearly reveals a stepwise progression of invasive melanoma from benign nevi (Fig. 5M), thus validating the reliability of our histology‐based nevus and melanoma staging (Figs 3, 4, 5). Together, our data indicate that targeted disruption of P53 at the DNA binding domain led to nevus formation and a stepwise evolution of invasive melanoma in *X. tropicalis*.

### 3.3 *tp53* ^*+/Δ7*^dysplastic nevus exhibits loss of heterozygosity

**Fig. 6:**
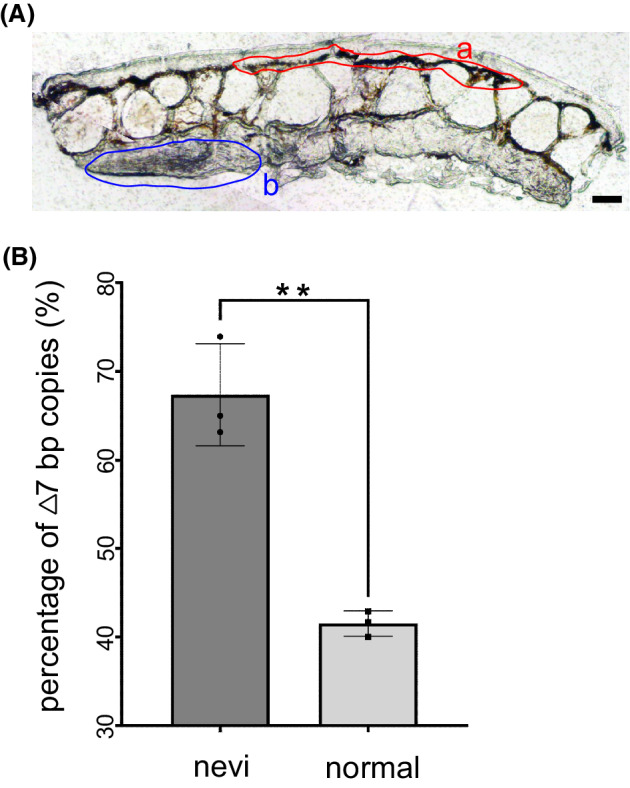
Loss of heterozygosity occurred in dysplastic nevus of a *tp53* ^*+/Δ7*^ mutant. (A) A 20 μm thick cryosection of the dysplastic nevus from a *tp53* ^*+/Δ7*^ mutant shows areas for laser capture microdissection‐mediated collection of lesion cells (a) and non‐lesion cells (b). Scale bar, 50 μm. (B) Histograms of Sanger DNA sequencing data (mean values ± SD) from three independent PCR amplifications show the occurrence of LOH in lesion cells. graphpad prism 9 (GraphPad Software) was used for the *t*‐test (**P < 0.01).

*TP53* loss of heterozygosity (LOH) can be frequently detected in tumor samples of human LFS patients [35] and tumors developed in mouse *Trp53* [36] and zebrafish *tp53* [37] heterozygotes. To determine whether LOH occurred in dysplastic nevi of the *tp53* ^*+/Δ7*^ frogs, pigmented nevus cells and neighboring stratum compactum cells were isolated by laser capture microdissection from a dysplastic nevus with a diameter of 2 mm for DNA extraction, PCR amplification, and Sanger DNA sequencing (Fig. 6A). The sequencing data revealed that LOH did occur in the dysplastic nevus cells but not in the neighboring tissue (Fig. 6B), indicating that the occurrence of *tp53* LOH is highly conserved in vertebrates and the nevus formation in this frog model is due to the loss of P53 activity. To further confirm the genotype–phenotype specificity and to rule out potential off‐target effects of CRISPR/Cas9, we identified genome‐wide potential off‐target sites with less than four mismatches for SpCas9 *tp53* targeting site (Table S3), of which we selected all the three with two mismatches for Sanger DNA sequencing analysis (for primer sequences, see Table S1) with genomic DNA extracted from the *tp53* ^*Δ7/Δ7*^ nevi. None of them shows any mutations (Fig. S3), suggesting low off‐target effects in this scenario. For the P53 family members P63 and P73, we were unable to detect any *tp73* transcripts in *tp53* ^*Δ7/Δ7*^ skin. No mutations were detected in four different *tp63* mRNAs expressed in *tp53* ^*Δ7/Δ7*^ nevi (Table S4). Together, our data indicate that it was the disruption of canonical full‐length P53 protein that resulted in the formation of nevi and melanomas in the *tp53* Δ7 mutant frogs.

### 3.4 Spontaneous formation of sarcomas and pancreatic ductal adenocarcinoma in *tp53* ^*Δ7*^ ^*/Δ7*^ frogs

**Fig. 7:**
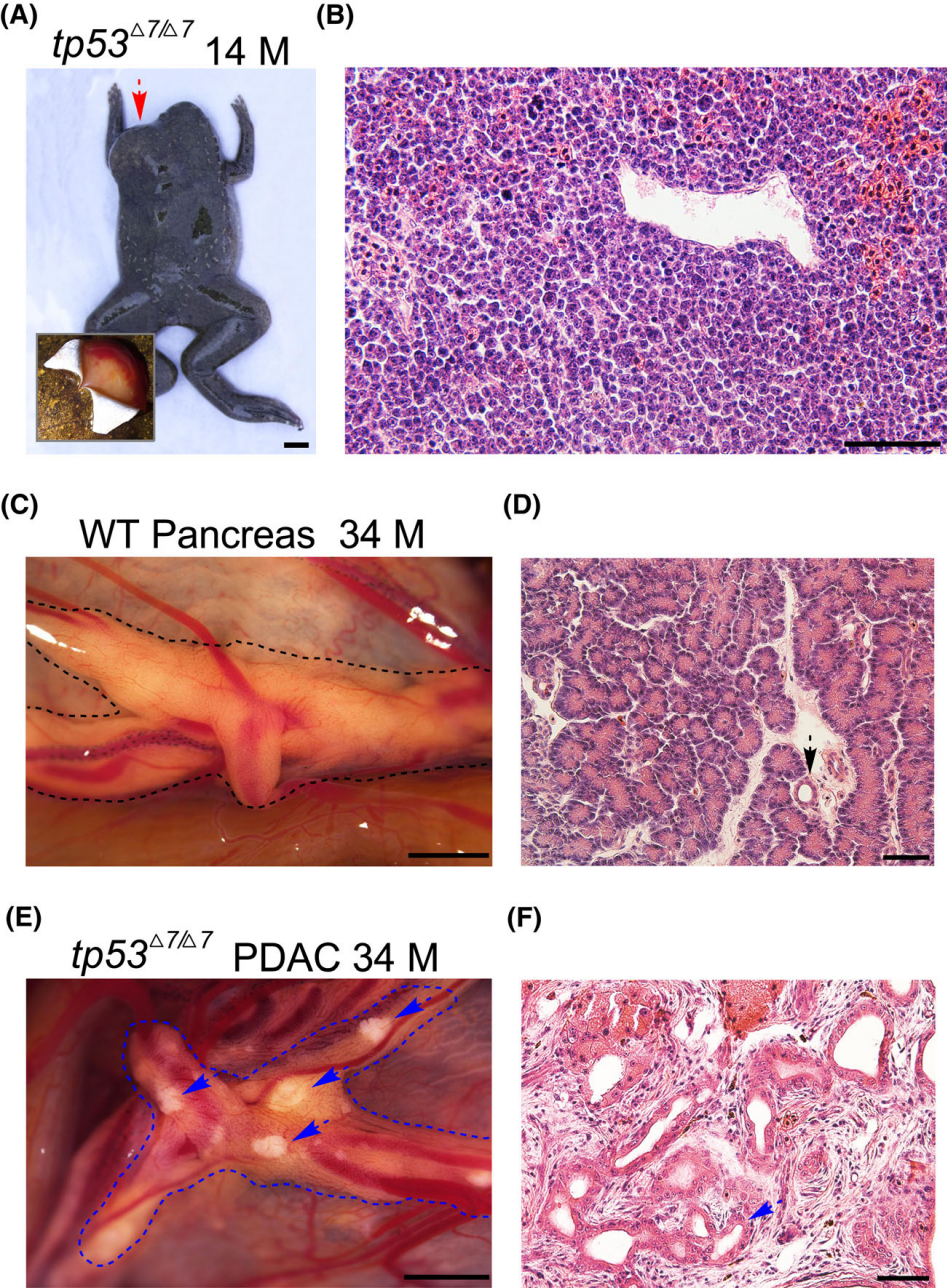
Round cell sarcomas and pancreatic ductal adenocarcinoma were developed in a *tp53* ^*Δ7/Δ7*^ adult frogs. (A) Dorsal view of the 14‐month‐old *tp53* ^*Δ7/Δ7*^ frog shows the location of the sarcoma (red dashed arrow). Dissection of the overlying skin revealed that the sarcoma grew from the hypodermis (bottom‐left corner). Scale bar, 5 mm. (B) Representative H&E staining of the sarcoma sections revealed histopathologic characteristics resembling human round cell sarcoma [38]. Similar structure was observed in all 50 serial sections. Scale bar, 50 μm. (C) Representative morphology of a dissected fresh pancreas from a 34‐month‐old wild‐type *Xenopus tropicalis* frog. Two frogs were dissected and similar pancreas morphology was observed. (D) Representative histology of the pancreas shown in C. Dashed arrow points to a normal pancreatic duct. Similar structure was observed in all 50 serial sections. (E) Morphology of a dissected pancreas from a 34‐month‐old *tp53* ^*Δ7/Δ7*^ frog. Dashed blue arrows highlight the light deposits that are not seen in wild‐type healthy pancreas. (F) Representative histopathology of the lesions seen in E shows neoplastic ducts (dashed blue arrow) embedded in fibrous tissue resulted from severe desmoplastic reaction, thus recapitulating the histopathologic characteristics of human pancreatic ductal adenocarcinoma [39]. Similar structure was observed in all 50 serial sections. Scale bars in C and E, 1 mm. Scale bars in D and F, 50 μm. M, months; WT, wild‐type.

Among the same 115 14‐month‐old *tp53* ^*Δ7/Δ7*^ frogs, 3 (about 3%) developed conspicuous subcutaneous sarcomas (Fig. 7A and data not shown). Dissection of one of the lesions revealed that the sarcoma grew from hypodermis with no attachment to the overlying skin (Fig. 7A). Based on histological data, the sarcoma is classified as a round cell sarcoma (Fig. 7B [38]). Thus, the *tp53* ^*Δ7/Δ7*^ frog line recapitulates one of the five core LFS cancers.

For internal tumor inspection, six frogs from the Clutch 1 *tp53* ^*Δ7/Δ7*^ homozygotes were sacrificed at the age of 34 months. Only one frog showed light deposits in pancreas, which could not be seen in wild‐type healthy pancreas (Fig. 7C,E). No tumors were found in other five frogs. Histological analyses revealed that the lesions possessed typical histopathological characteristics of human pancreatic ductal adenocarcinoma [39]. The structure of the regular distribution of intralobular ducts and acini seen in the wild‐type pancreas was completely destroyed in the lesions, in which neoplastic ducts were embedded in fibrous tissue due to severe desmoplastic reaction (Fig. 7D,F). Thus, the *tp53* ^*Δ7/Δ7*^ frog line also recapitulates human pancreatic ductal adenocarcinoma.

## 4 Discussion

In this study, upon stringent genotyping and phenotyping, including genotyping at both DNA and protein levels, X‐ray irradiation, off‐target analysis, LOH analysis, and histology‐based nevus/melanoma staging with RT‐PCR validation, we have provided several lines of evidence demonstrating the establishment of a canonical full‐length P53‐deficient *X. tropicalis* line that develops nevi and melanomas in addition to recapitulating some of the LFS cancers. In our model, the latency for invasive melanoma formation is relatively long and its penetrance is also low. In line with this, in a rare case, a single, advanced melanoma was indeed observed in one out of over 500 *tp53* ^−/−^ fish aged 11 months [16]. Given the hairy skin of mice and the zebra pattern of zebrafish integument, to phenotype potential cutaneous nevi and non‐invasive melanomas in any P53 modified mouse or zebrafish models, more careful inspection should be given, as the size of those nevi and melanomas might be too small to be easily missed. In contrast to our finding, no melanoma was described upon targeted disruption of *X. tropicalis* P53 at the proline‐rich domain [19]. This phenotypic discrepancy suggests that an intricate P53‐isoform network be conserved in *X. tropicalis*. It remains a challenge to define the consequence of any *tp53* alteration, including the Δ7 lesion, to the whole landscape of the P53 network in *X. tropicalis*.

In comparison with the total tumor incidence in *Trp53*‐disrupted mice, which is estimated at 74% by 6 months and increases over time [8, 40], the current overall tumor incidence observed in *tp53* Δ7 mutants is much lower. The survival rates of *tp53* Δ7 heterozygotes and homozygotes in the first 2 years are almost similar to that of wild‐type siblings. The average size of dysplastic nevi gradually increased over time and the melanoma incidence was also slowly increasing. As the life span of *X. tropicalis* (over 10 years, [19]) is significantly longer than that of mice (2 years), we speculate that *X. tropicalis tp53* Δ7 mutants might develop more invasive melanomas and other types of cancers in a relatively rapid pace at ages above 4 years. Nevertheless, these mutants are valuable for further investigation of the molecular mechanism underlying melanoma progression upon germline alteration of the *tp53* locus, such as if and how an order of further genetic alterations occurs.

## 5 Conclusions

In this study, we show that targeted disruption of *tp53* at the DNA binding domain in *X. tropicalis* predisposed the frogs to nevi and melanomas in addition to soft tissue sarcomas and pancreatic ductal adenocarcinoma. The latter belongs to the cancer types seen in LFS patients. Our data suggest that more dermatologic control should be given to LFS patients, which likely will settle the association of melanoma with LFS in epidemiology. The *tp53* Δ7 mutants are also valuable for further investigation of the molecular mechanism underlying melanoma progression upon germline alteration of the *tp53* locus.

## Conflict of interest

The authors declare no conflict of interest.

## Author contributions

RR, ZS, and YC conceived the project. RR and LL performed the experiments and analyzed the data together with GL, HJ, JH, WC, LF, and TX. ZS generated the *tp53* knockout founder frogs. RR and YC wrote the manuscript with input from all the authors.

### Peer review

The peer review history for this article is available at https://publons.com/publon/10.1002/1878‐0261.13301.

## Supporting information


**Fig. S1.** Invasive melanoma developed in a 20‐month‐old *tp53* ^*Δ7/Δ7*^ frog, as shown in Fig. 4.
**Fig. S2.** Gradually increase of the average size of dysplastic nevi over time.
**Fig. S3.** Sanger DNA sequencing data for three potential CRISPR/Cas9 off‐target sites.

**Table S1.** The primers (from 5′ to 3′ end) used in this project.
**Table S2.** Statistics on X‐ray irradiation.
**Table S3.** Potential off‐target sites predicted by CRISPOR.
**Table S4.** cDNA sequencing data for four different *tp63* mRNAs from *tp53* ^*Δ7/Δ7*^ nevi.

## Data Availability

The data that support the findings of this study are available on request from the corresponding author.
