# Supplementary material for: Disruption of tp53 leads to cutaneous nevus and melanoma formation in Xenopus tropicalis
Source: Mol Oncol. 2022 Aug 18;16(19):3554–67. doi: 10.1002/1878-0261.13301 (PMC9533689; doi:10.1002/1878-0261.13301)
Supplement: Supplementary file 1 — Fig. S1. Invasive melanoma developed in a 20‐month‐old tp53 Δ7/Δ7 frog, as shown in Fig. 4. Fig. S2. Gradually increase of the average size of dysplastic nevi over time. Fig. S3. Sanger DNA sequencing data for three potential CRISPR/Cas9 off‐target sites. [file MOL2-16-3554-s002.pdf]

Supplementary Figures for

Disruption of *tp53* leads to cutaneous nevus and melanoma formation in *Xenopus tropicalis*

Rensen Ran†, Lanxin Li†, Zhaoying Shi†, Guanghui Liu, Hao Jiang, Liangchen Fang, Tingting Xu, Jixuan Huang, Weiqi Chen, and Yonglong Chen*

*Correspondence to: chenyl@sustech.edu.cn

This PDF file includes:

Figs.S1 to S3

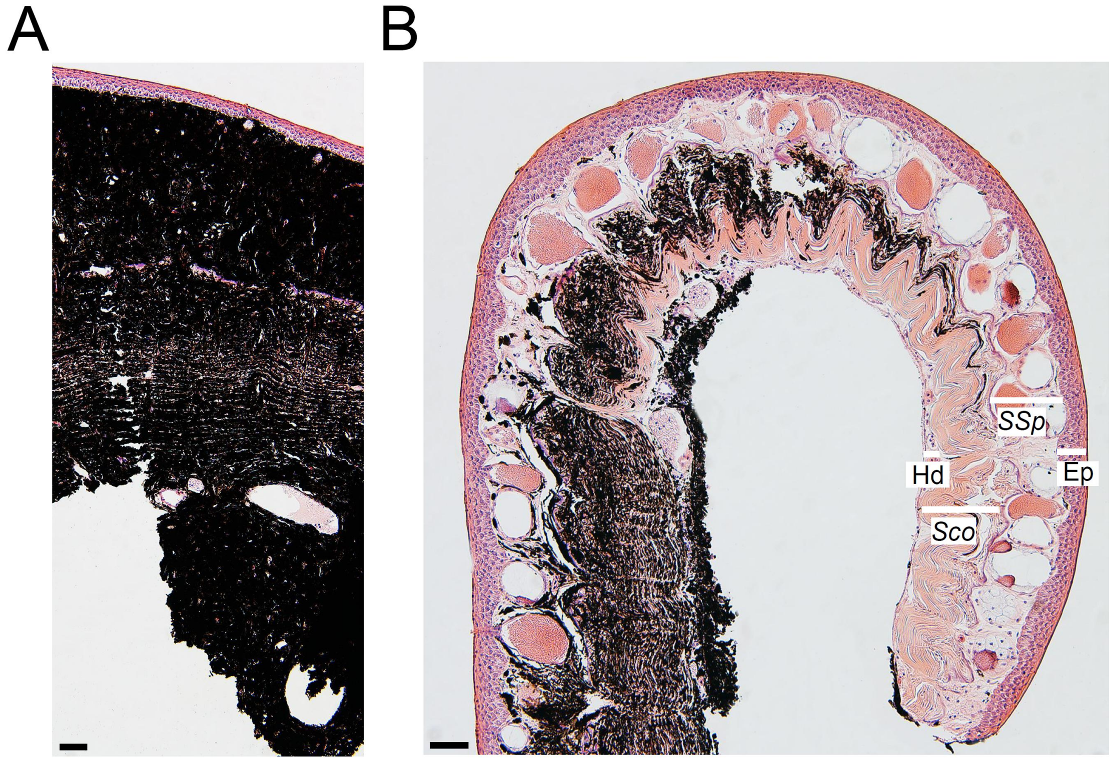

Fig. S1. Invasive melanoma developed in a 20-months old *tp53*$^{\Delta7/\Delta7}$ frog, as shown in Fig. 4. Scale bars, 50 μm.

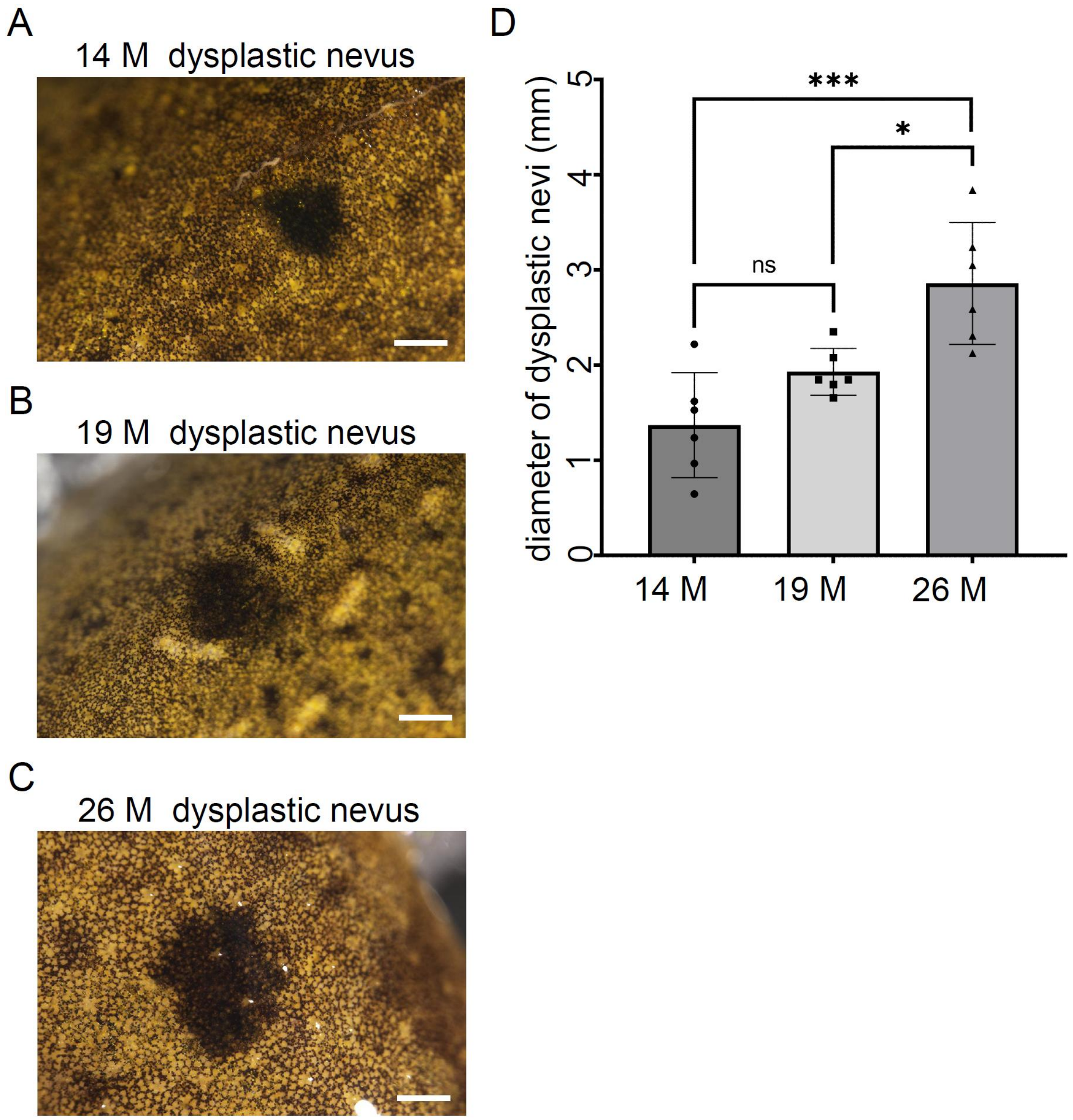

Fig. S2. Average size of the dysplastic nevi in *tp53*$^{\Delta7/\Delta7}$ frogs increased over time. (A-C) Representative morphology of the lesions. Scale bars, 1 mm. **(D)** Statistics (mean values ± SD) on the diameter measurements selectively for dysplastic nevi in *tp53*$^{\Delta7/\Delta7}$ frogs. Benign nevus, dysplastic nevus, and melanoma in situ can be distinguished by their darkness and shape alive. GraphPad Prism 9 was used for the t-test (* $p < 0.05$, *** $p < 0.001$, ns, no significant difference). M, months.

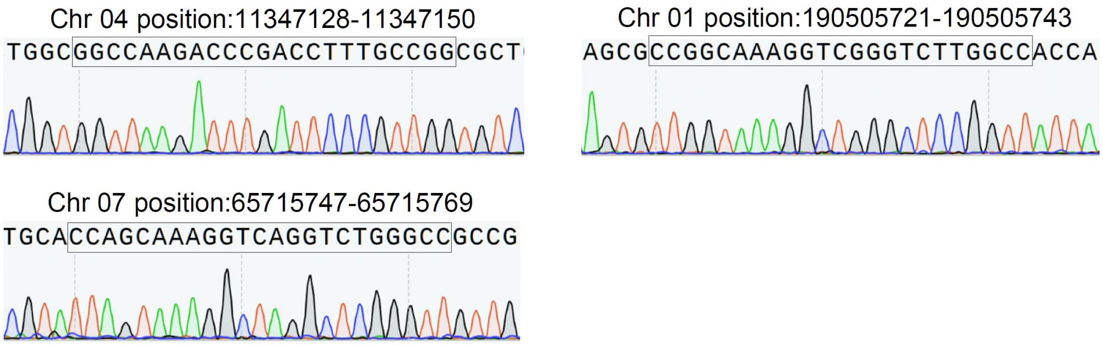

Fig. S3. Sanger DNA sequencing data show no mutations in the three potential off-target loci predicted by CRISPOR.
